# Spontaneous pneumomediastinum and pneumopericardium in a young healthy adult with plans for air travel

**DOI:** 10.1002/ccr3.3339

**Published:** 2020-09-18

**Authors:** Joseph Winterton, Simon Biart

**Affiliations:** ^1^ Royal Liverpool University Hospital Liverpool UK; ^2^ Acute Medical Unit Arrowe Park Hospital Wirral UK

**Keywords:** acute medicine, cardiothoracic surgery, respiratory medicine

## Abstract

Although rare, pneumomediastinum and pneumopericardium should be considered in patients presenting with sudden onset post‐tussive chest discomfort.

## Graphical abstract

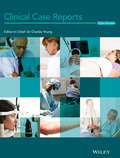

## 1 SUMMARY

A young healthy male presented with sudden onset chest and neck pain with dyspnea and dysphonia following a coughing spell. Examination revealed surgical emphysema in the left side of the neck and the left supraclavicular fossa. He had a tachycardia but was not hypoxic. A chest X‐ray confirmed surgical emphysema in the left side of the neck and raised concern of pneumomediastinum. The subsequent CT scan identified both pneumomediastinum and, more surprisingly, pneumopericardium. No pneumothorax was identified. The patient remained well throughout a 48‐hour period of observation and was managed conservatively, he was counseled against air travel at discharge.

## 2 CASE PRESENTATION

A 23‐year‐old man presented to the emergency department with sudden onset post‐tussive pleuritic chest pain and associated left‐sided neck pain. He complained of dyspnea in addition to rhinolalia—a symptom of the voice gaining a higher pitch. He had three days of coryzal symptoms prior. He had no past medical history or contributing family history, took no regular medications, and was not known to have any allergies. He did not smoke or use illicit substances for recreational use, drank minimal alcohol, and was physically active. On examination, the gentleman was tall with a medium build. On admission, he was in mild discomfort with a tachycardia but was hemodynamically stable and had no hypoxia or respiratory distress. There was no JVP elevation or muffling of heart sounds to suggest tamponade. He had surgical emphysema palpable in the left supraclavicular fossa, but had a nondeviated trachea and lung fields clear to auscultation with normal resonance.

## 3 INVESTIGATIONS

**Figure 1:**
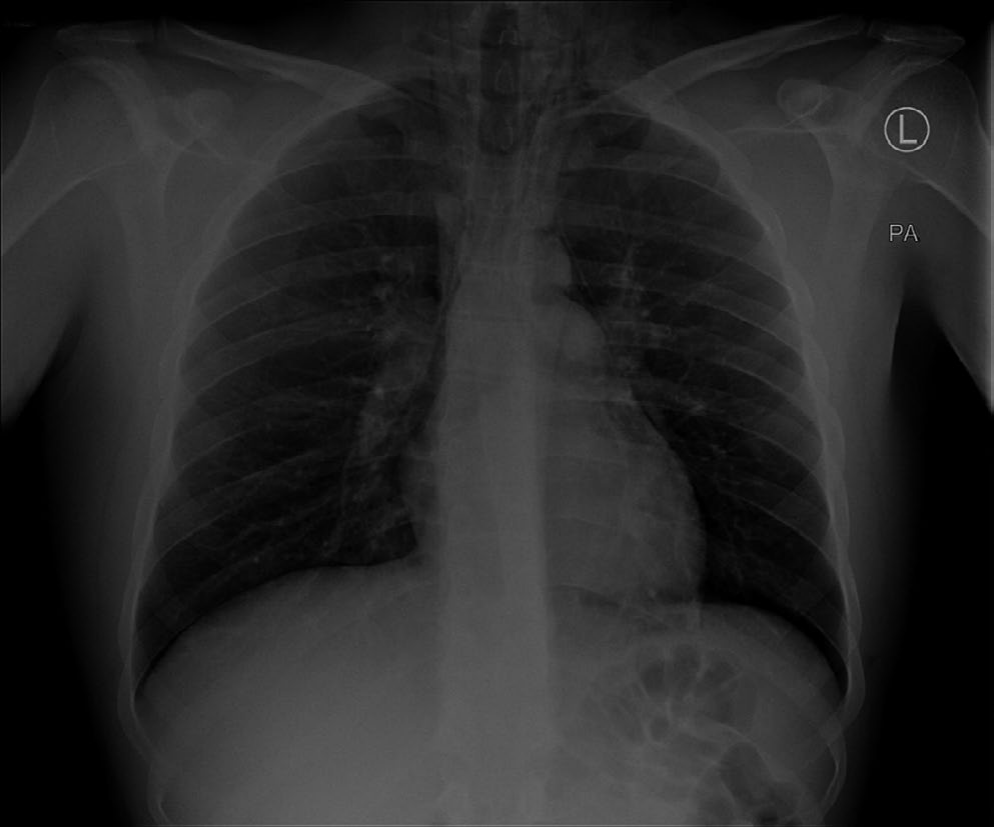
Admission chest X‐ray demonstrating pneumomediastinum and surgical emphysema in the left supraclavicular fossa

**Figure 2:**
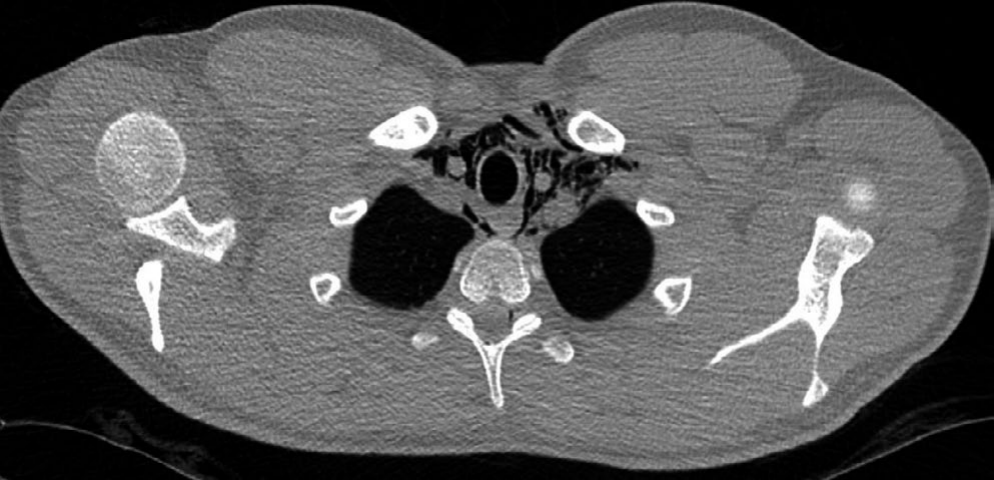
CT scan demonstrating pneumomediastinum

**Figure 3:**
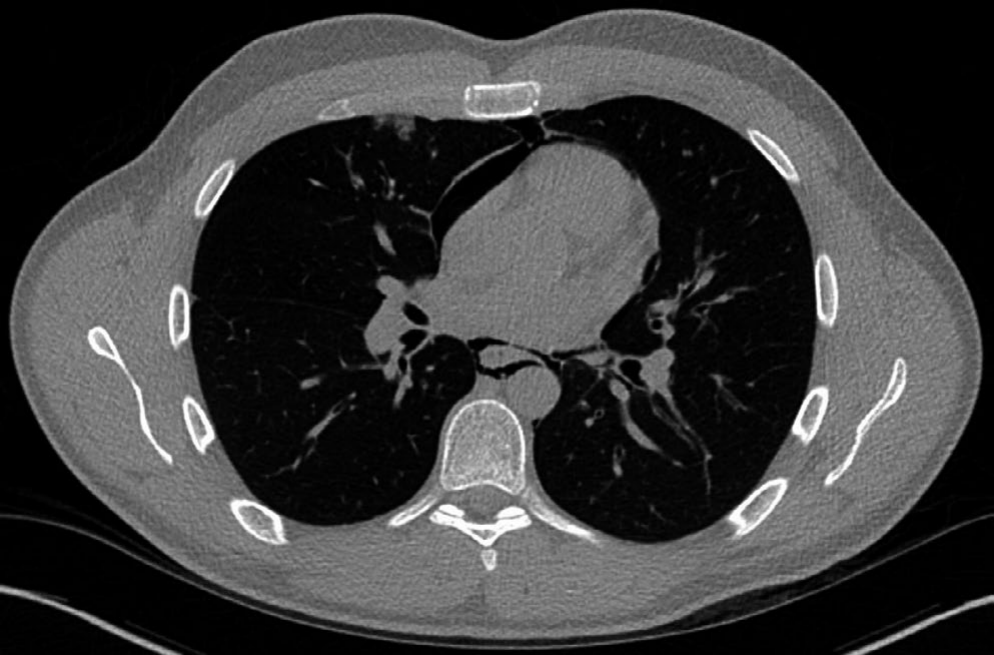
CT scan demonstrating pneumopericardium

The patient's white cell count was 13 with a mild neutrophilia. An ECG showed a sinus tachycardia. A chest X‐ray identified surgical emphysema extending into the neck from the left lung apex with associated pneumomediastinum (Figure 1). An urgent CT with contrast esophagogram was performed. This not only confirmed the presence of surgical emphysema and pneumomediastinum (Figure 2), but also revealed pneumopericardium (Figure 3). No identifiable source for the free air was identified.

## 4 DIFFERENTIAL DIAGNOSIS

With a presentation of sudden onset pain, dyspnea, and surgical emphysema, a pneumothorax was the primary differential. Pulmonary embolism was unlikely in the absence of clotting risk factors or specific clinical features such as hemoptysis or hypoxia. The radiological suggestion of pneumomediastinum was unexpected, but the dysphonia and rhinolalia made this diagnosis more probable. Once identified, a source for the pneumomediastinum and pneumopericardium was sought. A traumatic cause had been excluded by the history. CT scan ruled out Boerhaave esophageal rupture, soft tissue infection, or lung pathology. Other cases of isolated pneumopericardium published have been related to perforated gastric ulcers, but this young man had no symptoms or signs suggestive of this.^1^ A potentially life‐threatening sequelae of free air in the pericardial space is tension pneumopericardium resulting in tamponade; however, the patient remained hemodynamically stable with no clinical evidence of this, and so, no echocardiogram was performed.

## 5 TREATMENT

The patient was admitted and treated with adequate analgesia. Given he had a productive cough and raised inflammatory markers, he was started on oral antibiotics for an infectious bronchitis. The case was discussed with cardiothoracic specialists. Following the review of the CT scan, it was suggested that the most probable cause of the presentation was a ruptured pulmonary bleb resulting from forceful coughing. Although usually attributed to the formation of spontaneous pneumothorax, this was thought to be the origin of the pneumomediastinum and pneumopericardium. The parent team was advised to treat conservatively and monitor for a further 24 hours to ensure the surgical emphysema was not increasing in size—which would suggest ongoing air leak. In the absence of pneumothorax or hypoxia, oxygen therapy was not utilized.

The patient was observed for a period and discharged with advice to return if symptoms worsened. The patient was counseled regarding the lifetime contraindication to scuba diving and the need to avoid flying until resolution of free air was confirmed at follow‐up.^2^

## 6 OUTCOME AND FOLLOW‐UP

The admission was a frustrating one for the patient, who despite feeling well‐advised to remain in for observation initially. After discharge, follow‐up was arranged with respiratory physicians with repeat chest X‐ray. Although a plain chest X‐ray can normally be used to see resolution of a pneumothorax or even pneumomediastinum, this would not adequately ensure resolution of pneumopericardium and further CT imaging would be necessary. The patient experienced ongoing discomfort and shortness of breath initially after discharge, but this soon settled.

The patient had extensive travel plans arranged for the weeks following discharge. These had to be postponed on medical advice. Careful communication of the potential sequelae of air travel was required in addition to advice on a life‐long avoidance of scuba diving.

## 7 DISCUSSION

A MEDLINE search with the terms "spontaneous pneumomediastinum" and "pneumopericardium" returned 40 results. Of these, 11 were truly case reports of adults with spontaneous pneumomediastinum with associated spontaneous pneumopericardium. Cases with such mild symptoms and such significant radiological findings are rare. It is suggested that this pathology could be misdiagnosed or missed with ease in a young, healthy population.

Spontaneous pneumomediastinum (SPM) is defined as free air within the mediastinum, without an identifiable cause. The condition may be referred to as Hamman syndrome, following the first reported case in 1939.^3^ To be classed as spontaneous, clear extrinsic causes must be ruled out; most frequently recognized are esophageal rupture (Boerhaave's), and infection with gas‐producing organisms and trauma. The dissection of alveolar air through the interstitium of the lung, along the bronchovascular sheath to the hilum, is known as the Macklin effect.^3^, ^4^ Ruptured alveoli can result from increased bronchoalveolar pressure. This phenomenon is attributed to a range of causes, including the hyperventilation of asthma or those in diabetic ketoacidosis, performing the Valsalva maneuver, substance inhalation, or, as in this case, vigorous coughing.^5^, ^6^, ^7^, ^8^

SPM affects predominantly males aged 17‐25 and has an estimated incidence of 1 in 30 000 hospital admissions.^9^ Patients with SPM will most commonly present complaining of chest pain and shortness of breath with associated subcutaneous emphysema of the neck.^10^ CT scan including an oral contrast study to rule out esophageal rupture is suggested as the favored method of investigation, given 70% of SPM is missed on plain chest X‐ray.^5^ Cases of spontaneous pneumopericardium in conjunction with pneumomediastinum are extremely rare.

Pneumopericardium, although requiring higher pressures to develop than SPM, is most likely caused by air dissecting through the lung interstitial and medially along the venous sheaths into the pericardial space.^11^ There is no clinical guideline for the management of SPM or pneumopericardium. Both conditions can follow a benign course, suggested treatment being with conservative measures such as analgesia and observation in addition to appropriate investigation of the cause.^11^ In the conservative management of pneumothoraces, supplemental oxygen is utilized to increase gas reabsorption—some literature suggests oxygen can be used for a similar purpose in SPM. ^12^

Both pneumopericardium and pneumomediastinum may be complicated by tension that may require surgical intervention.^12^ Rarely, simple pneumomediastinum can progress into a tension pneumomediastinum. This occurs with significant accumulation of air in the mediastinum due to the persistent air leak of a missed esophageal or pulmonary defect.^12^ Although identified exclusively only in cases of traumatic pneumopericardium,^13^ tension pneumopericardium was a possible sequelae in this case. This is a life‐threatening condition leading to cardiac tamponade and risk of hemodynamic instability and arrest. Tension pneumopericardium should therefore be excluded once the diagnosis of free air in the pericardial space is made. Hemodynamic instability clearly raises suspicion. ECG has been suggested as beneficial in early recognition of this diagnosis.^14^ Nonspecific ST and T wave changes and decreased amplitude on ECG have been recognized in at least two cases of tension pneumopericardium.^15^, ^16^

There is a lack of guideline or evidence for the management of pneumomediastinum and pneumopericardium; therefore, British Thoracic Society (BTS) guidelines on pneumothorax were adapted to form the basis of treatment, counseling, and follow‐up^2^—the risks of tension and tamponade from rapid pressure change theoretically remain the same.

## 8 LEARNING POINTS/TAKE‐HOME MESSAGES

- Pneumomediastinum and pneumopericardium should be a consideration in adult patients presenting with sudden onset chest pain—the transient and mild nature of symptoms in young people do not correlate well with the severity of pathological findings on radiological imaging and may contribute to underdiagnosis.
- The management of spontaneous pneumomediastinum and pneumopericardium is largely conservative, but clinicians should be vigilant for life‐threatening sequelae of tension pneumomediastinum and tension pneumopericardium.
- Although there is a lack of guidance for this specific condition, clinicians should be clear in communicating risk with regard to air travel and other restricted activities, in this case adapted from BTS pneumothorax guidelines. The impact of this diagnosis on a patient can be restrictive, and counseling should be careful with adequate consideration of patient concerns.

## CONFLICT OF INTEREST

The authors have no conflict of interests to declare.

## AUTHOR CONTRIBUTIONS

JW: researched and prepared the manuscript and was involved in patient care. SB: involved in patient care, provided guidance, and edited the final manuscript.

## ETHICAL APPROVAL

Written informed consent was obtained from the patient for publication of this case report.

## CONSENT STATEMENT

Published with written consent of the patient.
